# Supplementary material for: A biomimetic 2D transistor for audiomorphic computing
Source: Nat Commun. 2019 Aug 1;10:3450. doi: 10.1038/s41467-019-11381-9 (PMC6673702; doi:10.1038/s41467-019-11381-9)
Supplement: Supplementary file 1 — Supplementary Information [file 41467_2019_11381_MOESM1_ESM.docx]

Supplementary Information

A Biomimetic 2D Transistor for Audiomorphic Computing

Sarbashis Das^1^, Akhil Dodda^2^, and Saptarshi Das^2, 3,*^

*^1^Electrical Engineering, Pennsylvania State University, University Park, PA 16802*

*^2^Engineering Science and Mechanics, Pennsylvania State University, University Park, PA 16802*

*^3^Material Research Institute, Pennsylvania State University, University Park, PA 16802*

**Supplementary Note 1**

*Discussion on Virtual Source (VS) Model:* In the VS model, both the subthreshold and the above threshold behavior is captured through a single semi-empirical and phenomenological relationship that describes the transition in channel charge ($Q_{CH}$) from weak to strong inversion. We have used a modified VS model to capture the variation in the electrostatic potential along the width of the channel as described in Eq. S1.

$$I_{DS}=\frac{V_{DS}}{R_{CH}+{2R}_{A}}; R_{CH}=\frac{L_{CH}}{\mu_{N}Q_{CH}}; Q_{CH}=\int_{0}^{W_{CH}} C_{G}m\frac{k_{B}T}{q}\log\left[ 1+\exp\left( \frac{V_{CH}(x)-V_{T}}{\frac{mk_{B}T}{q}} \right) \right]dx; [S1]$$

In Eq. S1,$R_{CH}$ is the channel resistance corresponding to the split-gated region and$R_{A}$ is the access resistance due to the rest of the ungated region as shown in the right panel of Fig. 2j. Further, $L_{CH}=4 \mu m$ is the channel length,$W_{CH}=2 \mu m$ is the channel widths, and $\mu_{N}$ is the carrier mobility for electrons in MoS_2_ which was extracted from the peak transconductance value and was found to be 46 cm^2^/V-s. The band movement factor, $m$, was obtained from the subthreshold slope ($SS=mk_{B}Tln10$) of the experimental transfer characteristics of the fully top gated device and was found to be 13, the threshold voltage,$V_{T}$ was found to be -12V, and$R_{A}$ was found to be 1.19 MΩ-μm. Note, that in the subthreshold regime, the inversion charge increases exponentially with$V_{CH}$, whereas above threshold, the inversion charge is a linear function of$V_{CH}$, which is seamlessly captured through the VS model.

**Supplementary Note 2**

*Discussion on the Footprint of the Audiomorphic Device:* The audiomorphic MoS_2_ transistor is expected to provide significant area benefit when compared to functionally equivalent logic formed by NAND gates. This is because NAND logic gates in the CMOS technology consist of 4 FETs (2 PMOS and 2 NMOS) each with 3 terminals (source, drain and gate). Therefore, to match N_P_ split gate pairs the NAND CMOS circuit layout would require an area of ~12*N_P_*A, where, A is the layout area occupied by a single terminal corresponding to a given technology node. However, the layout for the analog multi-gate audiomorphic transistor would necessitate an area of ~ (2*N_P_+2)*A, which include the N_P_ split gate pairs and 1 source and 1 drain terminal. Clearly, area benefit becomes significant with increasing N_P_. However, this argument is rather simplistic and requires further in depth analysis.

**Supplementary Note 3**

*Discussion on the Scalability of the Audiomorphic Device:* The scalability or ultra-thin body MOSFET is captured through a simple parameter called the screening length ($\lambda_{SC}$), which is given by equation S2:

$$\lambda_{SC}=\sqrt{\frac{\varepsilon_{body}}{\varepsilon_{ox}}t_{body}t_{ox}} [S2]$$

Where,$t_{body}$ and$t_{ox}$ are the thicknesses and, $\varepsilon_{body}$ and$\varepsilon_{ox}$are the dielectric constants of the channel and the gate oxide, respectively. In order to avoid short channel effects the channel length of an FET (L_CH_) has to be at least three times higher than the screening length, i.e. L_CH_ > 3λ_SC_. For atomically thin semiconducting monolayers of 2D materials,$t_{body}$ ≈ 0.6 nm, which corresponds to$\lambda_{SC}$ ≈ 1.3 nm, whereas, for the most advanced FinFET technology the thickness of Si fin can be scaled down to only 5 nm without severely increasing the bandgap due to quantum confinement effects and reducing the mobility due to enhanced surface roughness scattering. Therefore, use of 2D material such as MoS_2_ allows for the geometric miniaturization without any loss of electrostatic integrity. In other word more split gate pairs can be fitted into the audiomorphic device for a given channel length of the MoS_2_ FET.

**Supplementary Note 4**

*Discussion on Axonal Conduction Velocity:* As described in the main text, classical cable theory can be used to describe the signal propagation through the axon by involving two critical parameters, the length constant ($\lambda$), which determines how far the action potential can propagate without regeneration, and, the time constant ($\tau$), which determines how fast the action potential can propagate, resulting in a finite axonal conduction velocity$v_{A}=\frac{\lambda}{\tau}$. The axonal conduction velocity is linearly proportional to the diameter of the myelinated axon and is found to vary greatly in the vertebrate nervous system. The fastest axons are ~ 20 μm in diameter and conduct at ~ 120 m/s, whereas, the slowest axons are ~ 0.1 μm in diameter and conduct at ~ 0.3 m/s.^1^ While rapid conduction is always desirable for speedy response of the nervous system, it comes at a relatively high price in the brain volume. For example, in the above-cited case, fastest axons are 40000X worse in area efficiency compared to the slowest axons. The nervous system deploys fast axons for the processing of sensory information that is critical for the survival of the species. For invertebrate axons, the absence of myelin sheath makes the axonal conduction even slower and necessitates a far more dramatic increase in the axonal diameter for rapid conduction of action potential. For example, squids have giant axons with diameter ~0.5 mm which are dedicated for generating their escape response against approaching predators.^2^ However, only a few of such giant axons can fit into the squid nervous system. In contrast, the vertebrate solution to speedy response is myelination of the axon allowing them to achieve conduction velocities comparable to those seen in giant squid axons, but occupying 10000X lesser brain volume. In the barn owl delay line axons only become myelinated when the head reaches its adult width.^3^ Intracellular recordings from the NM axons in NL reveal a conduction velocity of 3-5 m/s.^17^

**Supplementary Figure 1**

***Figure S1.*** *Transfer characteristics of fully top-gated MoS2 FET for different back gate voltages.*


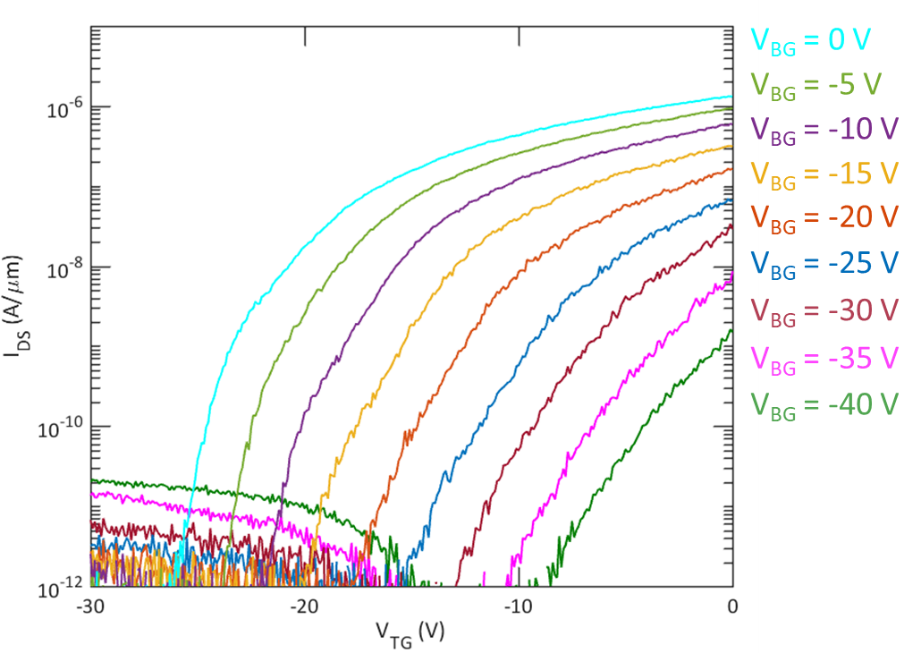


**Supplementary References**

1 Waxman, S. & Bennett, M. V. Relative conduction velocities of small myelinated and non-myelinated fibres in the central nervous system. *Nature New Biology* **238**, 217 (1972).

2 Cole, K. S. & Curtis, H. J. Electric impedance of the squid giant axon during activity. *The Journal of general physiology* **22**, 649-670 (1939).

3 Cheng, S. M. & Carr, C. E. Functional delay of myelination of auditory delay lines in the nucleus laminaris of the barn owl. *Developmental neurobiology* **67**, 1957-1974 (2007).
